# Supplementary material for: DACH1 mutation frequency in endometrial cancer is associated with high tumor mutation burden
Source: PLoS One. 2020 Dec 30;15(12):e0244558. doi: 10.1371/journal.pone.0244558 (PMC7773279; doi:10.1371/journal.pone.0244558)
Supplement: S1 File — (PDF) [file pone.0244558.s001.pdf]

April 23, 2020

M2GEN Bioinformatics Analysis Methods

Matched Germline and Tumor Analysis involves the following components:

i. Adapter trimming

Adapter sequences are trimmed from the raw sequencing FASTQ files. Adapter-trimming via via *k*-mer matching is performed along with quality-trimming and filtering, contaminant-filtering, sequence masking, GC-filtering, length filtering and entropy-filtering. The trimmed FASTQ files are used as input to the read alignment process.

ii. Read Alignment, sequencing quality metrics, marking duplicate reads and base recalibration

Matched WES G and T adapter-trimmed FASTQ files are aligned to the human genome reference (GRCh38/hg38) using the BWA-MEM aligner. Resulting alignment files are sorted, duplicate reads are marked, and base qualities recalibrated. Alignment files are compressed in .cram format, which can be converted to .bam format using SAMtools. Sequencing quality control metrics are calculated using Picard; generating Hybrid Selection specific metrics (hs_metrics), general alignment statistics, duplication metrics, and insert sizes (fragment lengths).

iii. Variant Detection and Variant Annotation

Using an alignment file from a normal/germline sample as input, germline single nucleotide variants (SNVs) and insertions/deletions (INDELs) are called using the Sentieon DNAseq algorithm, which matches GATK 4.1 best practices. Using alignment files from a pair of patient matched tumor and normal/germline samples, somatic SNVs and INDELs are called using Sentieon TNseq, which matches MuTect2 v4.0 without downsampling for higher accuracy and improved detection of variants. Variant files are provided in compressed Variant Call Format (.vcf.gz) and Genomic VCF (.g.vcf.gz) file formats. Germline and somatic VCF files are annotated to provide variant context beyond genomics coordinates and allele fraction. Functional annotation includes amino acid coding changes, association with the Catalogue of Somatic Mutations in Cancer (COSMIC), ClinVar relationships among human variants and phenotypes, the Genome Aggregation Database (gnomAD) of population polymorphisms, and familial cancer genes.

iv. Filtering

Somatic mutations within the VCF file are assessed for confidence via application of a true positive probability scoring system that reduce the False Discovery Rate (FDR). The probability filter employs a Fisher's Exact Test with a null hypothesis that for a given

somatic mutation, a patient's tumor and normal samples will have the same underlying allele fractions. Despite the difference in depth of coverage rates between the tumor and normal sequencing events, the distribution of alternate allele reads remains the same. One-tailed p-values are reported in the VCF INFO field with designation PROBF. If PROBF ≥0.05 (indicating null hypothesis not rejected) and normal alt reads >0, then the "probability_filter" tag is added to the FILTER field. For multiallelic locations, PROBF is currently reported as "NA" and the FILTER field is not modified.

Somatic mutations within the VCF file are also assessed for contaminating population polymorphisms and recurrent sequencing artifacts using a Panel of Normals (PoN). Position recurrent artifacts are usually indentified and removed during standard tumor/normal comparison; however, this process is dependent upon adequate coverage across the tumor/normal samples. Construction and application of a PoN identifies both population polymorphisms and locations prone to aberrant mapping or systematic sequencing artifacts. The M2GEN PoN is constructed from the AVATAR germline variants catalogue. The M2GEN PoN filter is applied by checking every reported somatic mutation against the AVATAR germline variants catalogue and adds "panel_of_normals" in FILTER field or replaces if FILTER is "PASS". The PoN filter tag is used to reduce the False Discovery Rate (FDR) for somatic mutations by identifying both population polymorphisms and systematic sequencing artifacts. The PoN includes germline variants that are present in >3% of the entire population of unrelated normal samples.

v. Microsatellite Instability (MSI)

Microsatellite Instabile (MSI) is a classification of mutations within microsatellite regions of the genome. There are two possible classifications for MSI status, MSI-High and Microstellite Stable (MSS). MSI-H means that there is a high amount of instability; associated with a buildup of somatic microsatellite mutations in tumor cells that can lead to a spectrum of molecular and biological changes including high tumor mutational burden. Tumors with MSI-H are sensitive to immune checkpoint blockade inhibitors (such as PD-1 and PD-L1 inhibitors). MSS implies a low degree of mutation within microsatellite regions. An MSI score is calculated as follows: the number of MSI sites with somatic mutation divided by all valid MSI sites. Where all valid sites are defined as a subset of all microsatellite regions with sequencing coverage exceeding a minimum threshold. It is recommended that an MSI score cutoff value of 20% be used to define MSI-H (MSI-H: MSI score >= 20%; MSS: MSI score < 20%).

vi. Tumor Mutational Burden (TMB)

TMB is calculated using the count of non-synonymous somatic mutations (single nucleotide variants and small insertions/deletions; including missense, stop gain, stop loss and start loss mutations) per mega-case in the coding region of the specific capture kit.

RNAseq Tumor Pipeline Analysis is processed according to the workflow outlined below using GRCh38/hg38 human genome reference sequencing and GenCode build version 32.

i. Adapter trimming

Adapter sequences are trimmed from the raw tumor sequencing FASTQ file. Adapter-trimming via via *k*-mer matching is performed along with quality-trimming and filtering, contaminant-filtering, sequence masking, GC-filtering, length filtering and entropy-filtering. The trimmed FASTQ file is used as input to the read alignment process.

ii. Read Alignment

The tumor adapter-trimmed FASTQ file is aligned to the human genome reference (GRCh38/hg38) and the Gencode genome annotation v32using the STAR aligner. The STAR aligner generates multiple output files used for Gene Fusion Prediction and Gene Expression Analysis.

iii. Gene Fusion Prediction

STAR-Fusion and Arriba Gene Fusion algorithms are applied to the STAR aligner output files. Gene Fusion predictions from both STAR-Fusion and Arriba are merged into a single output file that removes duplicate putative gene fusion calls, removes putative gene fusion calls of low confidence – reporting gene fusions with at least one (1) junction read and at least one (1) spanning read, and removes gene fusion calls occurring within the same gene, within SnoRNAs, within rRNAs, or mitochondrial genes – which are areas considered to be contributing to high false-positive rate and generally uninformative. Additional heuristics are applied to the merged putative fusion calls to coalesce to the most dominate gene isoform in a set of reported putative gene fusion calls that either share an identical breakpoint or within a set of overlapping putative gene fusion calls. Finally. Each of the merged putative gene fusions is represented in graphical output .pdf, where each tumor sample has a .pdf document where each page displays a single graphic representation of putative gene fusion (Fig. 1).

iv. RNA Expression

RNA expression values are calculated and reported using estimated mapped reads, Fragments Per Kilobase of transcript per Million mapped reads (FPKM), and Transcripts Per Million mapped reads (TPM) at both transcript level and gene level based on transcriptome alignment generated by STAR.
