# Supplementary material for: DACH1 mutation frequency in endometrial cancer is associated with high tumor mutation burden
Source: PLoS One. 2020 Dec 30;15(12):e0244558. doi: 10.1371/journal.pone.0244558 (PMC7773279; doi:10.1371/journal.pone.0244558)
Supplement: Attachment — Submitted filename: Response to Reviewers.docx [file pone.0244558.s002.docx]

**Response to Reviewers**

1. Please ensure that your manuscript meets PLOS ONE's style requirements, including those for file naming. The PLOS ONE style templates can be found at

[https://journals.plos.org/plosone/s/file?id=wjVg/PLOSOne_formatting_sample_main_body.pdf](https://nam04.safelinks.protection.outlook.com/?url=https%3A%2F%2Fjournals.plos.org%2Fplosone%2Fs%2Ffile%3Fid%3DwjVg%2FPLOSOne_formatting_sample_main_body.pdf&data=04%7C01%7Cmbri229%40uky.edu%7Cd1902d0e2b00468a515608d876cc961f%7C2b30530b69b64457b818481cb53d42ae%7C0%7C0%7C637389968346409284%7CUnknown%7CTWFpbGZsb3d8eyJWIjoiMC4wLjAwMDAiLCJQIjoiV2luMzIiLCJBTiI6Ik1haWwiLCJXVCI6Mn0%3D%7C1000&sdata=jrOqggam0PaPka4NHJtFOwD27anmtpVBeNevdWfVv6E%3D&reserved=0) and

[https://journals.plos.org/plosone/s/file?id=ba62/PLOSOne_formatting_sample_title_authors_affiliations.pdf](https://nam04.safelinks.protection.outlook.com/?url=https%3A%2F%2Fjournals.plos.org%2Fplosone%2Fs%2Ffile%3Fid%3Dba62%2FPLOSOne_formatting_sample_title_authors_affiliations.pdf&data=04%7C01%7Cmbri229%40uky.edu%7Cd1902d0e2b00468a515608d876cc961f%7C2b30530b69b64457b818481cb53d42ae%7C0%7C0%7C637389968346409284%7CUnknown%7CTWFpbGZsb3d8eyJWIjoiMC4wLjAwMDAiLCJQIjoiV2luMzIiLCJBTiI6Ik1haWwiLCJXVCI6Mn0%3D%7C1000&sdata=BIVvNYiRoUVfcWTRZLbwV3fGu2%2Be26Z9KR96ljc2x3E%3D&reserved=0)

The file naming and legends were updated according to the PLOS ONE style templates as requested. The title page was also updated according to the templates.

2. We note that Figure 1 in your submission contain copyrighted images. All PLOS content is published under the Creative Commons Attribution License (CC BY 4.0), which means that the manuscript, images, and Supporting Information files will be freely available online, and any third party is permitted to access, download, copy, distribute, and use these materials in any way, even commercially, with proper attribution. For more information, see our copyright guidelines: [http://journals.plos.org/plosone/s/licenses-and-copyright](https://nam04.safelinks.protection.outlook.com/?url=http%3A%2F%2Fjournals.plos.org%2Fplosone%2Fs%2Flicenses-and-copyright&data=04%7C01%7Cmbri229%40uky.edu%7Cd1902d0e2b00468a515608d876cc961f%7C2b30530b69b64457b818481cb53d42ae%7C0%7C0%7C637389968346409284%7CUnknown%7CTWFpbGZsb3d8eyJWIjoiMC4wLjAwMDAiLCJQIjoiV2luMzIiLCJBTiI6Ik1haWwiLCJXVCI6Mn0%3D%7C1000&sdata=yobhBvMbo%2FERxxXXyPMZtruCjJ%2BAQXHTWlMsZPWvKb0%3D&reserved=0).

We require you to either (1) present written permission from the copyright holder to publish these figures specifically under the CC BY 4.0 license, or (2) remove the figures from your submission:

2.2.    If you are unable to obtain permission from the original copyright holder to publish these figures under the CC BY 4.0 license or if the copyright holder’s requirements are incompatible with the CC BY 4.0 license, please either i) remove the figure or ii) supply a replacement figure that complies with the CC BY 4.0 license. Please check copyright information on all replacement figures and update the figure caption with source information. If applicable, please specify in the figure caption text when a figure is similar but not identical to the original image and is therefore for illustrative purposes only.

A replacement figure that complies with the CC by 4.0 license has been provided and uploaded as figure 1. M2Gen gives permission for the open-access journal PLOS ONE to publish the whole exome sequencing schematic image under the Creative Commons Attribution License (CCAL) CC BY 4.0. The permission form has been uploaded as requested as S2_File and the figure caption has been updated to reflect this permission.

 3. In your Methods section, please provide additional information about the participant recruitment method and the demographic details of your participants. Please ensure you have provided sufficient details to replicate the analyses such as: a) the recruitment date range (month and year), b) a description of any inclusion/exclusion criteria that were applied to participant recruitment, c) a description of how participants were recruited.

 These details were added to the section “study population” as requested.

4. In the ethics statement in the manuscript and in the online submission form, please provide additional information about the patient records used in your study, including: a) whether all data were fully anonymized before you accessed them; b) the date range (month and year) during which patients' medical records were accessed.

 Additional information was added regarding the interplay and contractual agreement that allowed access to the data between M2Gen/ORIEN Total Cancer Care, University of Kentucky, and the Kentucky Cancer Registry. This is further clarified in both the study design and study population sections.

5. Please provide the accession number and/or URL for any datasets obtained from the TCGA database.

The URL for the 586 patients accessed through the TCGA PanCancer Atlas database is listed here, including the endometrial cancer and carcinosarcoma patients: <https://www.cbioportal.org/results/cancerTypesSummary?cancer_study_list=ucec_tcga_pan_can_atlas_2018%2Cucs_tcga_pan_can_atlas_2018&Z_SCORE_THRESHOLD=2.0&RPPA_SCORE_THRESHOLD=2.0&data_priority=0&profileFilter=0&case_set_id=all&gene_list=DACH1&geneset_list=%20&tab_index=tab_visualize&Action=Submit>. This has also been included within the manuscript.

6. Thank you for stating the following in the Competing Interests section:

"I have read the journal's policy and the authors of this manuscript have the following competing interests: Dr. Piecoro’s spouse is employed by Exelixis, Inc. Dr. Hampton, is employed by M2Gen, a for profit company focused on providing oncology health informatics solutions to accelerate cancer treatment discovery, development, and delivery by leveraging clinical and molecular data."

We note that one or more of the authors are employed by a commercial company: M2Gen, Exelixis, Inc.

6.1. Please provide an amended Funding Statement declaring this commercial affiliation, as well as a statement regarding the Role of Funders in your study. If the funding organization did not play a role in the study design, data collection and analysis, decision to publish, or preparation of the manuscript and only provided financial support in the form of authors' salaries and/or research materials, please review your statements relating to the author contributions, and ensure you have specifically and accurately indicated the role(s) that these authors had in your study. You can update author roles in the Author Contributions section of the online submission form.

Please also include the following statement within your amended Funding Statement.

“The funder provided support in the form of salaries for authors [insert relevant initials], but did not have any additional role in the study design, data collection and analysis, decision to publish, or preparation of the manuscript. The specific roles of these authors are articulated in the ‘author contributions’ section.”

If your commercial affiliation did play a role in your study, please state and explain this role within your updated Funding Statement.

The funder provided support in the form of salaries for author OH, but did not have any additional role in the study design, data collection and analysis, decision to publish, or preparation of the manuscript. The specific roles of these authors are articulated in the ‘author contributions’ section.

Dr. Piecoro’s spouse’s employment by Exelixis, Inc, is noted but completely unrelated to this project. His funding organization did not play a role in the study design, data collection and analysis, decision to publish, or preparation of the manuscript in any way. Dr. Hampton is employed by M2Gen and serves as the chief officer of bioinformatics over the ORIEN pipeline. M2Gen did not play a role in the study design, data collection and analysis, or decision to publish. He and his department assisted in separating our patients into DACH1 mutated vs wild-type cohorts through the ORIEN software and provided the information regarding the ORIEN pipeline for this paper. Dr. Hampton was not involved in the subsequent bioinformatics analysis. He did provide final proofreading of the manuscript prior to submission.

This has been included in the cover letter as requested.

6.2. Please also provide an updated Competing Interests Statement declaring this commercial affiliation along with any other relevant declarations relating to employment, consultancy, patents, products in development, or marketed products, etc.

Within your Competing Interests Statement, please confirm that this commercial affiliation does not alter your adherence to all PLOS ONE policies on sharing data and materials by including the following statement: "This does not alter our adherence to  PLOS ONE policies on sharing data and materials.” (as detailed online in our guide for authors [http://journals.plos.org/plosone/s/competing-interests](about:blank)) . If this adherence statement is not accurate and  there are restrictions on sharing of data and/or materials, please state these. Please note that we cannot proceed with consideration of your article until this information has been declared.

The employment of OH by M2GEN does not alter our adherence to PLOS ONE policies on sharing data and materials.

Please include both an updated Funding Statement and Competing Interests Statement in your cover letter. We will change the online submission form on your behalf.

These are included.

Please know it is PLOS ONE policy for corresponding authors to declare, on behalf of all authors, all potential competing interests for the purposes of transparency. PLOS defines a competing interest as anything that interferes with, or could reasonably be perceived as interfering with, the full and objective presentation, peer review, editorial decision-making, or publication of research or non-research articles submitted to one of the journals. Competing interests can be financial or non-financial, professional, or personal. Competing interests can arise in relationship to an organization or another person. Please follow this link to our website for more details on competing interests: [http://journals.plos.org/plosone/s/competing-interests](about:blank)

7. In your Data Availability statement, you have not specified where the minimal data set underlying the results described in your manuscript can be found. PLOS defines a study's minimal data set as the underlying data used to reach the conclusions drawn in the manuscript and any additional data required to replicate the reported study findings in their entirety. All PLOS journals require that the minimal data set be made fully available. For more information about our data policy, please see [http://journals.plos.org/plosone/s/data-availability](https://nam04.safelinks.protection.outlook.com/?url=http%3A%2F%2Fjournals.plos.org%2Fplosone%2Fs%2Fdata-availability&data=04%7C01%7Cmbri229%40uky.edu%7Cd1902d0e2b00468a515608d876cc961f%7C2b30530b69b64457b818481cb53d42ae%7C0%7C0%7C637389968346429272%7CUnknown%7CTWFpbGZsb3d8eyJWIjoiMC4wLjAwMDAiLCJQIjoiV2luMzIiLCJBTiI6Ik1haWwiLCJXVCI6Mn0%3D%7C1000&sdata=aYyiK4vtWcQ8ol4KVAsepo8I1cI6FLNSe3lWXfPv9ac%3D&reserved=0).

Upon re-submitting your revised manuscript, please upload your study’s minimal underlying data set as either Supporting Information files or to a stable, public repository and include the relevant URLs, DOIs, or accession numbers within your revised cover letter. For a list of acceptable repositories, please see [http://journals.plos.org/plosone/s/data-availability#loc-recommended-repositories](https://nam04.safelinks.protection.outlook.com/?url=http%3A%2F%2Fjournals.plos.org%2Fplosone%2Fs%2Fdata-availability%23loc-recommended-repositories&data=04%7C01%7Cmbri229%40uky.edu%7Cd1902d0e2b00468a515608d876cc961f%7C2b30530b69b64457b818481cb53d42ae%7C0%7C0%7C637389968346429272%7CUnknown%7CTWFpbGZsb3d8eyJWIjoiMC4wLjAwMDAiLCJQIjoiV2luMzIiLCJBTiI6Ik1haWwiLCJXVCI6Mn0%3D%7C1000&sdata=lJokifCghCFR%2BY8%2BWSJEzaRlwIIgbOL%2BDMWaQPrvv%2F4%3D&reserved=0). Any potentially identifying patient information must be fully anonymized.

Important: If there are ethical or legal restrictions to sharing your data publicly, please explain these restrictions in detail. Please see our guidelines for more information on what we consider unacceptable restrictions to publicly sharing data: [http://journals.plos.org/plosone/s/data-availability#loc-unacceptable-data-access-restrictions](https://nam04.safelinks.protection.outlook.com/?url=http%3A%2F%2Fjournals.plos.org%2Fplosone%2Fs%2Fdata-availability%23loc-unacceptable-data-access-restrictions&data=04%7C01%7Cmbri229%40uky.edu%7Cd1902d0e2b00468a515608d876cc961f%7C2b30530b69b64457b818481cb53d42ae%7C0%7C0%7C637389968346439263%7CUnknown%7CTWFpbGZsb3d8eyJWIjoiMC4wLjAwMDAiLCJQIjoiV2luMzIiLCJBTiI6Ik1haWwiLCJXVCI6Mn0%3D%7C1000&sdata=k46xTeXq9p4rG2lEHhqrN6fybYea2bT2dB1992HUB44%3D&reserved=0). Note that it is not acceptable for the authors to be the sole named individuals responsible for ensuring data access.

The authors did not receive any special privileges in accessing the data. Due to the ethical and legal restrictions of public sharing of the genomic data for the patients, the data must be accessed through the third party broker at the Kentucky Cancer Registry. Raw data cannot be shared because they are both potentially identifying and contain sensitive patient data, including geographic location, dates of diagnosis and dates of testing and receiving a medication. In addition, there are contractual agreements between the University of Kentucky and the Kentucky Cancer Registry precluding data sharing. Any requests for data must be submitted to the KCR at:

Jacyln K. McDowell, Epidemiologist, Kentucky Cancer Registry

2365 Harrodsburg Rd, Suite A230

Lexington, KY 40504

859-218-2228

[Jaclyn.mcdowell@uky.edu](mailto:Jaclyn.mcdowell@uky.edu).

We will update your Data Availability statement to reflect the information you provide in your cover letter.

8. Please upload a copy of Supporting Information Appendix 1 which you refer to in your text on page 8 and 37.

This has been included.

==============================================================================

**Review Comments to the Author**

Please use the space provided to explain your answers to the questions above. You may also include additional comments for the author, including concerns about dual publication, research ethics, or publication ethics. (Please upload your review as an attachment if it exceeds 20,000 characters)

Reviewer #1: The title of this paper is DACH1 mutation frequency in endometrial cancer is associated with high tumor mutation burden. Based on that title a couple required elements are missing and pieces are out of place. Endometrial cancer, immediately thinking subsets (see major point 4). These subsets are somewhat addressed in table 6 (and are a confounder of table 3) but need to be flushed out in more detail and much earlier in the manuscript. The TCGA has MSI information in the unrestricted access clinical tables so this comparison is doable. This was included as requested. It's possible DACH1 doesn't fit into neatly into those subsets and that could justify all the pathway analysis but right now I wonder why the pathway analysis is there. I'd like to see a much deeper dive into analyses that support the title of the paper (e.g. the mutations in DACH1 or TMB by DACH1 status and also analyzed within subgroups). What's reported is technically correct but it lacks a greater context.

Major points:
1) Data availability, the authors should state what data is available in addition to the current statements about what data is not available.
This has been addressed above. Data must be requested through the Kentucky Cancer Registry as described as they are the third party honest broker due to the sensitivity and legal implications of using such data.

2) The bioinformatics method section only describes in very general terms the RNAseq methodology. Is Figure 1 copied from a brochure? Ideally would describe with references and version numbers. False discovery rate method for Figure 3 should be described here as well. Considering most of the figures are bioinformatics analyses, this section needs to be strengthened significantly.
Figure 1 is not copied from a brochure but is provided by M2Gen to depict their bioinformatics pipeline with additional details described in supplemental appendix 1.

3) Table 2 is screaming for comparison of MSS to MSI-H, ah there it is in Table 6.
This has been added as requested.

4) In comparing to TCGA there needs to be some description or normalization of cohorts. What fraction of each cohort is POLE, MSI-H, and other (copy number high vs low can be a judgement call so lets ignore it). Then what fraction of POLE, MSI-H and other is DACH1 mutated. This flaw permeates the manuscript and especially figure 8 since the TMB covariate is not controlled for.
Table 2b was added to address these concerns. Table 3 was updated to reflect and allow for normalization of the cohorts.

5) There are few enough DACH1 mutations that they should all be listed with protein effects. Are these nonsense alterations? Splicing? Silent? Are the amino acid changes consistent with loss of function (e.g. charge switch)?
This has been added as an additional table.

Minor points:
1) Line 194: p = 0.053 is approaching significance. Saying it is marginally significant makes me think the p value is 0.04999.
This is reworded to further clarify.

2) Figure 2 would look better as a lollipop plot. If the data is formatted properly, cBioportal will make one for you.
This has been changed to a lollipop plot as requested.

3) Figure 3 would benefit from labeling some of the genes called out in the text.
This has been updated to include the genes called out in the text as requested.

4) Figures 4, 5, 6 have copyright QIAGEN all rights reserved on them. Are the authors allowed to publish these plots in PLOS ONE?
The QIAGEN software was published by our department, allowing us the rights to publish the plots of our data using their software.

Reviewer #2: The authors have done a thorough job of doing all the relevant data analysis and the manuscript has also been well written. I have no additional comments on the paper and I recommend that the manuscript be accepted.

No additional requests noted from Reviewer #2.

6. PLOS authors have the option to publish the peer review history of their article ([what does this mean?](https://nam04.safelinks.protection.outlook.com/?url=https%3A%2F%2Fjournals.plos.org%2Fplosone%2Fs%2Feditorial-and-peer-review-process%23loc-peer-review-history&data=04%7C01%7Cmbri229%40uky.edu%7Cd1902d0e2b00468a515608d876cc961f%7C2b30530b69b64457b818481cb53d42ae%7C0%7C0%7C637389968346449260%7CUnknown%7CTWFpbGZsb3d8eyJWIjoiMC4wLjAwMDAiLCJQIjoiV2luMzIiLCJBTiI6Ik1haWwiLCJXVCI6Mn0%3D%7C1000&sdata=bi%2FwXG%2B0f%2FC0ttnYVW2hM6SMJQB2T5H8op5XayqLMB4%3D&reserved=0)). If published, this will include your full peer review and any attached files.


If you choose “no”, your identity will remain anonymous but your review may still be made public.


**Do you want your identity to be public for this peer review?** For information about this choice, including consent withdrawal, please see our [Privacy Policy](https://nam04.safelinks.protection.outlook.com/?url=https%3A%2F%2Fwww.plos.org%2Fprivacy-policy&data=04%7C01%7Cmbri229%40uky.edu%7Cd1902d0e2b00468a515608d876cc961f%7C2b30530b69b64457b818481cb53d42ae%7C0%7C0%7C637389968346449260%7CUnknown%7CTWFpbGZsb3d8eyJWIjoiMC4wLjAwMDAiLCJQIjoiV2luMzIiLCJBTiI6Ik1haWwiLCJXVCI6Mn0%3D%7C1000&sdata=xJ1V1n7CDMdA%2BQZAbJL2vkqBsNOxDqmq7hpeuT69i5M%3D&reserved=0).

Reviewer #1: No

Reviewer #2: No

[NOTE: If reviewer comments were submitted as an attachment file, they will be attached to this email and accessible via the submission site. Please log into your account, locate the manuscript record, and check for the action link "View Attachments". If this link does not appear, there are no attachment files.]

While revising your submission, please upload your figure files to the Preflight Analysis and Conversion Engine (PACE) digital diagnostic tool, [https://pacev2.apexcovantage.com/](https://nam04.safelinks.protection.outlook.com/?url=https%3A%2F%2Fpacev2.apexcovantage.com%2F&data=04%7C01%7Cmbri229%40uky.edu%7Cd1902d0e2b00468a515608d876cc961f%7C2b30530b69b64457b818481cb53d42ae%7C0%7C0%7C637389968346459254%7CUnknown%7CTWFpbGZsb3d8eyJWIjoiMC4wLjAwMDAiLCJQIjoiV2luMzIiLCJBTiI6Ik1haWwiLCJXVCI6Mn0%3D%7C1000&sdata=%2FWgdQQixlvEqUYBdmKmcScbmiwxWv4juygd47jDcC0Y%3D&reserved=0). PACE helps ensure that figures meet PLOS requirements. To use PACE, you must first register as a user. Registration is free. Then, login and navigate to the UPLOAD tab, where you will find detailed instructions on how to use the tool. If you encounter any issues or have any questions when using PACE, please email PLOS at [figures@plos.org](mailto:figures@plos.org). Please note that Supporting Information files do not need this step.
